# In Situ Synthesis of a Double-Layer Chitosan Coating on Cotton Fabric to Improve the Color Fastness of Sodium Copper Chlorophyllin

**DOI:** 10.3390/ma13235365

**Published:** 2020-11-26

**Authors:** Zhong Zhao, Chris Hurren, Mingwen Zhang, Liming Zhou, Jihong Wu, Lu Sun

**Affiliations:** 1 Institute for Frontier Materials, Deakin University, Geelong 3220, Australia; zhaozho@deakin.edu.au (Z.Z.); christopher.hurren@deakin.edu.au (C.H.); annie.zhang@deakin.edu.au (M.Z.); 2 School of Textile Science and Engineering, Wuhan Textile University, Wuhan 430073, China; 3 R&D Center, Guangdong Esquel Co. Ltd. Group, Foshan 528500, China; zhoulm@esquel.com

**Keywords:** chitosan, cross-linking, sodium copper chlorophyllin, bio-mordant

## Abstract

Natural dye’s poor affinity for cotton and poor fastness properties still hinder its applications in the textile industry. In this study, a doubled-layered chitosan coating was cured on cotton fabric to serve as bio-mordant and form a protective layer on it. Under the optimal treatment conditions, the maximum *q_e_* (adsorption amount) of the natural dye sodium copper chlorophyllin (SCC) calculated from the Langmuir isothermal model was raised from 4.5 g/kg to 19.8 g/kg. The dye uptake of the treated fabric was improved from 22.7% to 96.4% at 1% o.w.f. dye concentration. By a second chitosan layer cured on the dyed fabric via the cross-linking method, the wash fastness of the cotton fabric dyed with SCC can be improved from 3 to 5 (ISO 105 C-06). The natural source of the biopolymer material, chitosan, and its ability to biodegrade at end of life met with the initial objective of green manufacturing in applying natural dyes and natural materials to the textile industry.

## 1. Introduction

As green manufacturing is gradually becoming a norm, natural dyes, which are perceived as alternatives to less environmentally friendly oil derived synthetic dyes, such as azo dyes [1], have seen a revival in the textile dyeing industry [2]. Despite the advantages of natural dyes, their disadvantages, such as poor affinity for the substrates [3] and color fastness [4] still hinder the application of natural dyes in the textile industry.

**Figure 1:**
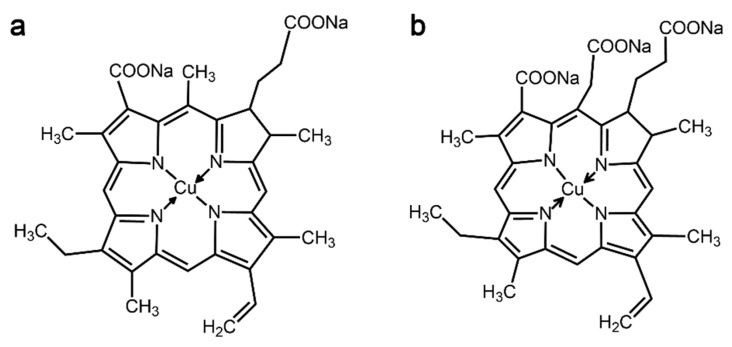
Two major components of SCC: (**a**) disodium copper chlorin e4 and (**b**) trisodium copper chlorin e6.

Sodium copper chlorophyllin (SCC), a green natural dye derived from chlorophyll [5] and also known as C.I. Natural Green 3, has been used as a food or beverage colorant in food processing [6,7]. It mainly contains two major components, disodium copper chlorin e4 and trisodium copper chlorin e6, whose molecular structures are presented in Figure 1. With the resurgence of natural dyes in the textile industry, their applications as natural dyes for silk [8] and cotton [9] have also been reported. However, due to the unstable chemical properties of porphyrin structures in SCC [10], textiles dyed with SCC have poor wash fastness and light fastness. To expand the applications of SCC to dyeing textiles, the color fastness of fabrics dyed with SCC should be improved.

Mordants have been used to enhance the dyeing performances of natural dyes since ancient times [11]. Mordants are used both to increase the affinity of dyes for fibers and improve their fixation within the fibers. Metal ions, such as copper, aluminum, iron and chromium, can improve the dye exhaustion by forming insoluble complexes with negatively charged groups of natural dye molecules, such as carboxylate ions and sulfonic groups [12]. However, these metal ions may pose threats to the environment once they are discharged into outer waters [13].

To address the adverse effects of metal ions, another mordanting method, cationic modification [14,15], was developed. Cationic agents, such as 3-chloro-2-hydroxypropyltrimethyl ammonium chloride (CHPTAC) [16], act as bridges to connect the dye molecules with the substrates by forming covalent bonds with the substrate via the epoxy groups in their molecules, and electrostatic interactions between the protonated amine groups in their molecules and the negatively charged groups of the dye molecules [17]. Unlike metal ions, which are toxic and cannot be easily removed from the dyeing wastewater [18], cationic agents can be completely degraded before the discharge. However, the cationic modification agents may also cause damage to the environment and the person who handles them, as these agents contain epoxy groups and long carbon chains, and thus have unpleasant smells [19].

As alternatives to cationic modification, bio-mordants derived from natural polymer materials have been applied in the textile dyeing [20,21,22]. In recent years, natural polymers, such as chitosan and sodium alginate, have been widely used in antibacterial finishes [23], micro-capsules [24], biocompatible drug delivery [25] and textile dyeing, due to their biocompatibility [26] and friendliness to the environment [27]. Among these bio-mordants, chitosan is a biopolymer derived from the shells of shrimp and crabs [28]. In most cases the chitosan is derived from shell waste products from the seafood industry [29]. Reusing it in the textile industry is also consistent with the initial aim of dyeing textiles with natural dyes.

Chitosan contains primary amines which can be positively charged after protonation [30]. It can be used to adsorb anionic ions in the wastewater via electrostatic interactions. It has been applied to the textile industry due to this property. Arimoto-Kobayashi et al. [31] have proved that chlorophyllin is absorbed onto the surface of chitosan under acidic conditions. Alonso et al. [32] have reported a method of cross-linking chitosan onto cotton fabric via using citric acid as the cross-linking agent and proved the antibacterial effects of chitosan-cross-linked cotton fabric. Mehrparvar et al. [33] used chitosan as a bio-mordant in wool dyeing and found that chitosan improved the exhaustion of the natural dye cochineal. In addition to the application of absorbing negatively charged substances, chitosan can also be applied to finish textiles [34,35], e.g., the durable press finish of cotton [36] and an antibacterial finish [37], as it can form durable thin layers on the substrates via cross-linking methods [38] due to its high polymerization degree and flexibility [39].

**Figure 2:**
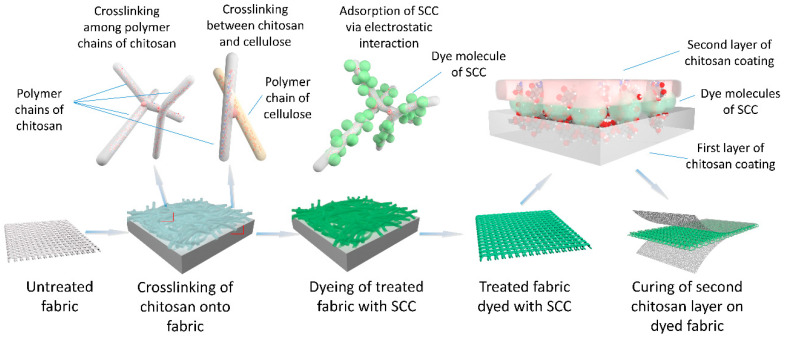
Schematic diagram of cross-linking of chitosan before and after dyeing.

In this study, chitosan was first cross-linked onto cotton fabric as a bio-mordant to improve dye exhaustion of SCC. Improved fixation of the dyed fabric was achieved by forming a cross-linked chitosan layer on the dyed fabric through the same cross-linking method with a high concentration chitosan solution employed. Figure 2 illustrates the processes of the treatment. Chitosan-cross-linked cotton was prepared by treating the cotton fabric with a chitosan solution at a low concentration. Adsorption of negatively charged SCC onto the treated fabric, which was positively charged at a pH < pKa of chitosan, was promoted in dyeing the cotton fabric with SCC solution. This electrostatic interaction resulted in the improvement of SCC exhaustion. Then, the dyed fabric obtained under the optimal treating conditions and dye concentration was further treated with chitosan solution with higher concentration to form a cured chitosan layer, which improved the color fastness of the dyed fabric.

An orthogonal experiment was first carried out to optimize the pre-treatment conditions and dye concentration. Then, another orthogonal experiment was conducted to find out the optimal post-treatment conditions. The adsorption kinetics and isotherms of SCC by the treated fabric and untreated control were investigated. Fabrics before and after the two cross-linking of chitosan processes were characterized. The handle of these samples was also evaluated.

## 2. Experimental

### 2.1. Materials

Chitosan powder with ≥95% deacetylation degree was purchased from Macklin Biochemical Co., Ltd. (Shanghai, China) and used as supplied. Analytical reagent grade (AR) citric acid monohydrate, trisodium citrate dihydrate, sodium perborate tetrahydrate and sodium carbonate were supplied by Sinopharm Chemical Reagent Co., Ltd. (Shanghai, China). Sodium copper chlorophyllin (also named C.I. Natural Green 3) was purchased from Tokyo Chemical Industry Co., Ltd. (Tokyo, Japan). Bleached single jersey knitted cotton fabric was supplied by the Esquel Group (Foshan, China). All the water used in this experiment was deionized water purified by a Milli-Q water purifying system (Darmstadt, Germany).

### 2.2. Cross-Linking of Chitosan

A diagram of the cross-linking of cotton fabric with chitosan via citric acid is presented in Figure S1. Chitosan solutions with gradient concentrations were separately prepared by dissolving 0.1–0.5 g (at an interval of 0.1 g) of chitosan in five 50 mL, 40 g/L citric acid solutions. In this study, citric acid not only acted as an acid to dissolve chitosan in an aqueous system under acidic conditions, but also a cross-linking agent to “bridge” chitosan and cellulose via esterification (Figure S5). Five 5.6 g pieces of undyed knitted cotton fabric (with a density of 180 g/m^2^) were immersed in each of the five as-prepared chitosan solutions. The excess chitosan solution was removed by a P-AO pad mangle (Yanuo Precision Machinery, China) to ensure 100 ± 5% liquid exhaustion. The setting of curing temperature was based on the findings from the study of Coma et al. on cross-linking of cotton via citric acid [40]. One-hundred and eighty degrees Celsius is an appropriate temperature that triggers the esterification of hydroxyl groups in cellulose and the carboxyl groups in citric acid [40]. The treated fabrics were first cured at 180 °C for 60 s with a HB-Q3000 heat-setting machine (Ludhiana Dyeing Machinery Works, Ludhiana, India), and then washed with 10 g/L sodium citrate solution and dried at 80 °C for 5 min.

### 2.3. Dyeing of Cross-Linked Fabric

The five fabric samples with different chitosan concentrations and one untreated control sample were dyed with SCC in an HTX-12 dyeing machine (Huaxia Technology, Jingjiang, China). A SCC solution was prepared following a dye concentration of 1% on weight of fabric (o.w.f.) and a solid to liquid ratio of 1:30. The fabric samples were separately placed in dyeing pot which contained the dye solutions. The dyeing pots were then heated from room temperature to 95 °C at a heating rate of 5 °C/min. The temperature was held at 95 °C for 1 h. The dyed fabrics were thoroughly rinsed with deionized water and dried in dark at room temperature. The K/S of each fabric sample was measured by a CS-650A spectrophotometer (CHNSpec Technology, Hangzhou, China) with a D65 light source and 10° observer (SCI mode). Another group of four fabrics treated with different conditions (as detailed in Table 1) were dyed with SCC under the same dyeing conditions and dye concentration. The K/S of the dyed fabrics were determined and compared to identify the critical factor that resulted in the improvement of K/S in the cross-linking of the cotton fabric. The ingredients used in each treatment of the samples are listed in Table 1.

### 2.4. Pre-Treatment Optimization and Dyeing

An orthogonal experiment, which consisted of 4 factors and 3 levels for each factor, was carried out to determine the optimal parameters for achieving maximum fixation of SCC. To minimize the detrimental effect of yellowing that may be caused by the curing process at high temperature, the curing time was minimized to the lowest level (no more than 100 s). Absorption kinetics of the fabric treated under the optimal pre-treatment conditions and the untreated control dyed with SCC at the optimal o.w.f. were calculated by determining the dye exhaustion at certain time intervals. The SCC exhaustion of the treated fabric and untreated control were calculated via Equation (1).

(1) $$\mathrm{SCC}\;\mathrm{exhaustion}\;\left(\%\right)=\frac{\left(A_{0}-A_{t}\right)}{A_{0}}\times 100\%$$

where A_0_ and A_t_ are the initial absorbance and the absorbance at time t of the dye bath at λ_max_ = 405 nm, respectively.

### 2.5. Adsorption Kinetics and Isotherms

Five grams of bleached plain knitted fabric with a density of 180 g/m^2^ was dyed with 1% o.w.f. SCC and a 30:1 liquid ratio at 95 °C for 1 h. The kinetics of SCC absorption on cotton fabric were investigated by monitoring the absorbance of the dye solution at certain time intervals. The dyeing process was carried out under continuous heat generated by an HBC-25 thermostated shaker bath (Huibao Dyeing and Finishing Machinery, Foshan, China). The absorbance of dye solution was scanned with a TU-1900 UV–Vis spectrophotometer (Purkinje General Instrument, Beijing, China).

Isotherms were investigated by dyeing the treated fabric and untreated control with a series of SCC solutions with gradient concentrations at 95 °C for 1 h. The absorbance of the SCC solution at λ_max_ = 405 nm was measured before and after the dyeing process. The molecular extinction coefficient of the SCC solution ε was 48.04 L·g^−1^·cm^−1^. The concentration of the SCC solution at the equilibrium of dyeing was calculated via the standard curve of SCC (shown in Equation (2)).

A = 48.04C(2)

where A is the absorbance of an aqueous solution of SCC at λ_max_ = 405 nm, and C is the concentration of the aqueous solution of SCC at the absorbance A.

### 2.6. Post-Treatment Optimization

A chitosan solution with a concentration of 30 g/L was used to treat the SCC-dyed fabric, which had the highest K/S among all the samples in the pre-treatment process. The pickup rate for the SCC-dyed fabric was set at 100 ± 5%. The treated SCC-dyed fabric was subjected to simulated sunlight radiation (with a wavelength range from 350 to 780 nm) generated by a 300 W PLS-SXE 300 Xenon lamp (Perfect Light, Beijing, China). The effect of cross-linking of chitosan on improving the color light fastness of the treated samples was investigated by measuring the color change (ΔE) before and after the radiation at an interval of 1 h. A piece of untreated SCC-dyed fabric was subjected to the same radiation as control. The wash fastness of the samples was tested following the ISO 105 C-06 standard.

Another orthogonal experiment which consisted of 3 factors and 3 levels of each factor was designed to investigate the impacts of chitosan usage, curing time and pick-up rate on the color fastness properties of the dyed fabric. All the samples were cured under the same conditions specified in Section 2.4. A piece of untreated SCC-dyed fabric was set as the control. Wash fastness of the treated fabrics was investigated according to the ISO 105 C-06 standard. Each sample was subjected to one wash cycle during each wash fastness test. The light fastness and the rubbing fastness of the samples were examined according to Method 3 in ISO 105 B-02 and ISO 105 X-12 respectively. The hydrophilic property the samples was characterized by measuring their contact angles with an FM40 EasyDrop contact angle measuring instrument (KRÜSS GmbH, Hamburg, Germany). ΔE of the treated fabrics was measured with the splash spectrophotometer. It was employed as an index to evaluate the color change caused by cross-linking under different conditions.

### 2.7. Characterization of Cross-Linked Cotton

Four samples, including the undyed greige fabric, sample 2 from the first orthogonal design before the dyeing process, sample 2 from the first orthogonal design after dyeing and sample 8 from the second orthogonal design after the treatment, were designated as greige fabric, pre-treated fabric, SCC-dyed fabric and post-treated fabric, respectively. All the four samples were characterized with SEM, FTIR, XRD and XPS to verify the cross-linking of chitosan via citric acid and reveal how cross-linking affected the properties of the cotton fabric. SEM images were obtained by scanning the samples with a Phenom Pro Desktop SEM (Thermo Fisher Scientific, Waltham, MA, USA). FTIR spectra were recorded with a Nicolet iS5 FTIR spectrometer (Thermo Fisher Scientific, Waltham, MA, USA). X-ray diffraction spectra were obtained by scanning the chitosan powder and the powder of the fabrics with Cu Kα radiation generated by a MiniFlex 600 X-ray diffractometer. X-ray photoelectron spectroscopy (XPS) was recorded with an AXIS ULTRA X-ray photoelectron spectrometer (Kratos Analytical Ltd., Manchester, UK). Loading chitosan onto the surface of fabric increased the weight and thickness of the fabric, resulting in deterioration of the fabric handle. The effect of cross-linking of chitosan on the fabric handle was an important index for evaluating the performance of the coating. In this study, a Wool HandleMeter (AWTA Ltd., Kensington, Australia) was used to determine the difference in handle of the fabrics after the two treatments. The handle tests were conducted following a laboratory standard reported by Naebe et al. [41]. Each fabric sample was cut into three circular specimens with a diameter of 100 mm. The mass of each specimen was measured with a balance. The mean mass of the three specimens was set as an input parameter for the test. The test results of each sample were exported after the tests of the three specimens with the HandleMeter. The flexibility properties of the four samples were also measured to provide further determination of how the cross-linking treatment affected the fabric’s mechanical properties. The flexibility test was carried out with a M507 fabric flexibility tester (Qingdao Shanfang Textile Instrument Co., Ltd., Qingdao, China) following the method reported by Wang et al. [42].

## 3. Results and Discussion

### 3.1. Pretreatment Optimization

**Figure 3:**
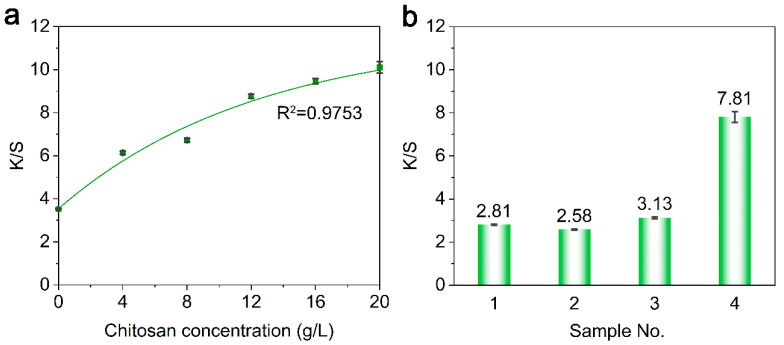
(**a**) K/S of fabrics treated with a chitosan solution with gradient concentrations; (**b**) K/S of fabrics treated with the chemicals detailed in Table 1.

The relationship between the chitosan usage and the K/S value of treated fabric was investigated by treating the knitted fabric with a series of chitosan solutions with gradient concentrations. The results were quantified by measuring each fabric’s K/S after dyeing under same dyeing conditions. The K/S values of the treated samples dyed with SCC are presented in Figure 3.

The K/S of the dyed fabric had a positive logarithmic relationship (R^2^ = 0.9753) with the concentration of chitosan solution applied to the fabric (Figure 3a). The K/S of Sample 4 in Figure 3b was doubled compared to the rest three samples. This confirms that only when chitosan was involved in the treatment could the K/S of the dyed fabric be improved, indicating that chitosan is the key ingredient which can improve the uptake of SCC.

The highest dye exhaustion (96.4%) was achieved with a 1% o.w.f. dye concentration on a 12 g/L chitosan pad solution with a 100 ± 5% pick-up cured at 180 °C for 60 s (Table 2). Curing time (Factor 3) had a significant effect on the dye exhaustion percentage of Sample 2. When the curing time was set at 80 s and 100 s, the corresponding dye exhaustion of treated samples lowered, compared with the samples treated at 180 °C for 60 s. The main reason was that curing at 180 °C may have led to the degradation of citric acid and chitosan, as the two substances start to melt at 153 °C [43] and 180 °C, respectively. The degradation of the two substances can be confirmed by the occurrence of yellowing on the surfaces of the samples curing at 180 °C for and 100 s (Figure S2).

### 3.2. Adsorption Kinetics and Isotherms

**Figure 4:**
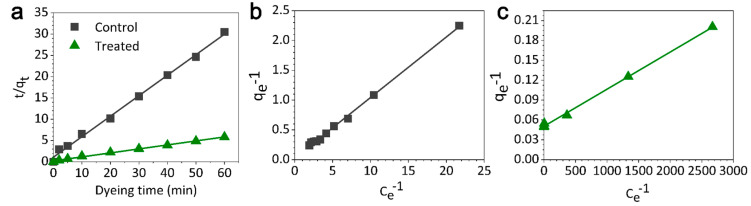
(**a**) Fitted curves of SCC on untreated control and treated cotton fabrics with the pseudo-second-order model, and Langmuir adsorption isotherms of SCC on the (**b**) untreated control and (**c**) pre-treated fabric.

Figure 4a shows the absorption kinetics of SCC onto the treated fabric and untreated control obtained by fitting the *t/q_t_* to dyeing time curve with the pseudo-second-order model. *t* is the dyeing time and *q_t_* is the weight of dye adsorbed onto the fabric per unit weight at time *t*.

The SCC adsorption kinetics of the treated fabric and untreated control both show good correlations with the pseudo-second-order model (*R^2^* treated: 0.9964 and untreated: 0.9970). The theoretical maximum adsorption of SCC absorbed by the samples with unit weight (*q_e_*_, *cal*_) calculated via the fitting equations (Equations (3) and (4)) for the untreated control and the treated fabric are 2.0 and 10.3 g/kg, respectively. The experimental maximum adsorption of SCC absorbed by the samples with unit weight (*q_e_*_, *exp*_) calculated with the input of the actual dye exhaustion for the untreated control and treated fabric are 2.0 and 10.3 g/kg, respectively.

t/qt = 0.4815 t + 1.0766(3)

t/qt = 0.0938 t + 0.2110(4)

The differences between the fitted results (*q_e_*_, *cal*_) and experimental data (*q_e_*_, *exp*_) for the untreated control and treated fabric [8] are both low, which in turn proves the capability of the fitted equations for predicting the maximum amount of SCC at the equilibrium of SCC absorption. In addition, the dye exhaustion by the treated sample was dramatically improved when compared with the untreated control as the equilibrium of SCC absorption was reached at a later time in the dyeing process.

Figure 4b,c show the Langmuir adsorption isotherms of the untreated control and treated fabric under the dyeing conditions of 95 °C for 1 h. The adsorption isotherms of SCC by control and treated fabrics can both be fitted with the Langmuir adsorption isotherm model (Equations (5) and (6)) with *R^2^* values of 0.9977 and 0.9983, respectively. The maximum *q_e_* (*Q*) of the cotton fabric, calculated using Equation (7), was improved from 4.5 to 19.8 g/kg after treatment with the optimal treating conditions.

*q_e_*^−1^ = 0.1015 Ce^−1^ + 0.0276 (5)

*q_e_*^−1^ = 5.6043 × 10^−5^ Ce^−1^ + 0.0504 (6)

(7) $$\frac{1}{q_{e}}=\frac{1}{Q}+\frac{1}{QbC_{e}}$$

where *q_e_* can *C_e_* are the dye amount and the concentration of dye solution at the equilibrium of adsorption, respectively; *Q* is the maximum amount of dye adsorbed by the fabric at the equilibrium; *b* is a constant of Langmuir isotherm.

### 3.3. Post-Treatment Optimization

**Figure 5:**
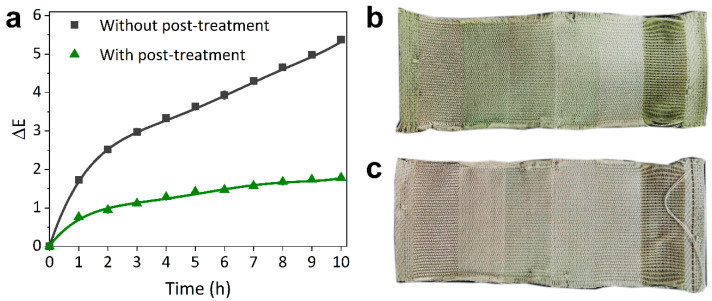
(**a**) ΔE of the SCC-dyed fabric with and without post-treatment caused by exposure to simulated sunlight radiation (with a wavelength range from 350 to 780 nm), and the multi-fiber reference fabric of the SCC-dyed fabric (**c**) with and (**b**) without post-treatment after the wash fastness test (ISO 105 C-06).

The light fastness of the SCC-dyed fabric treated with chitosan was better than that without treatment (Figure 5a) over all of the simulated sunlight irradiation times. This improvement can be attributed to a combination of the increase in depth of shade of the treated fabric (Table S2) and the protective effect of the chitosan layer formed on the fabric. The stained wash fastness standard multi-fiber fabric (Figure 5b,c) showed that dyed fabric being pre-treated with a 30 g/L chitosan solution and cured at 180 °C for 60 s improved wash fastness from 3 to 4–5. The improvement in wash fastness properties may be attributed to the mordant effect provided by the chitosan biopolymer, which was cross-linked onto the dyed fabrics via the cross-linking agent of citric acid.

The wash fastness of samples 1 and 8 reached 5 after curing at 180 °C for 100 s with a large loading amount of chitosan (Table 3). It is also notable that the gradient concentrations of chitosan solutions in this orthogonal experiment were higher than those in the first orthogonal experiment; thus, the influence of curing time on the wash fastness of the treated sample was opposite to that in the first orthogonal experiment. Curing at 180 °C for 60 or 80 s leads to incomplete cross-linking of chitosan onto the cotton fibers.

**Figure 6:**
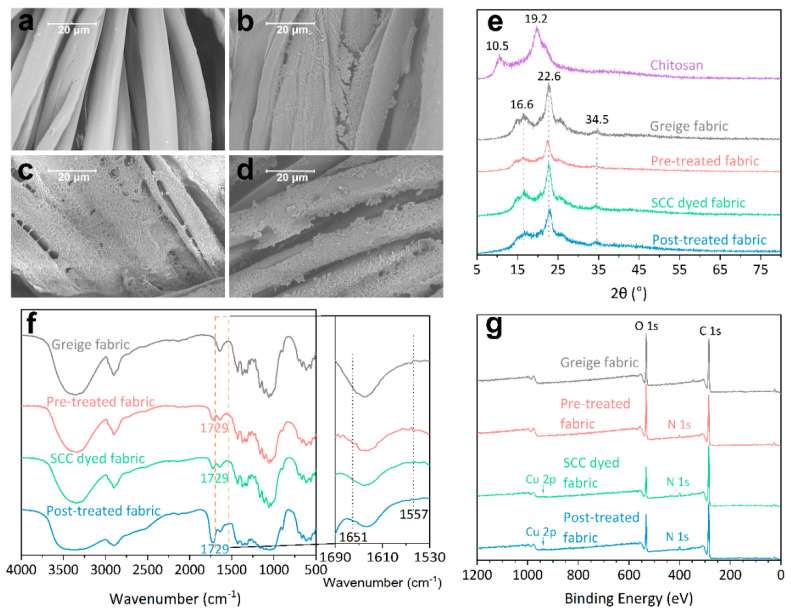
SEM images of (**a**) greige fabric, (**b**) pre-treated fabric, (**c**) SCC-dyed fabric and (**d**) post-treated fabric; and (**e**) XRD, (**f**) FTIR and (**g**) XPS wide-scan spectra of the four samples.

Contact angles of the post-treated fabrics (Figure S3) were measured after all the samples were rinsed with deionized water and dried at room temperature in the dark. The contact angles of all the treated samples varied within the range of 120° to 144°, indicating the surface of the fabric turned hydrophobic after chitosan cross-linking. It is notable that the contact angles of samples 4, 7 and 8 first increased and then decreased with the curing time. This change in contact angle was caused by the fact that exposure of the dyed fabric treated with chitosan solution to the high temperature environment will cause the degradation of chitosan. When the curing time was prolonged, the chitosan degraded and the chitosan layer formed on the cotton fabric was also damaged to a certain extent. This was then reflected by the result that the contact angle of the treated fabric declined. In addition, compared with sample 9, the contact angle of sample 7 decreased while increasing the concentration of chitosan solution at the same curing time of 80 s. This indicates that 30 g/L is not the optimal concentration of chitosan solution in the cross-linking process. The reason is that for a given size of the fabric, the number of binding sites on the cotton fabric for the esterification reaction is limited. In the pre-treatment process, the hydroxyl groups in the cotton fabric were already subjected to the esterification process. Therefore, increasing the concentration of the chitosan solution in the post-treatment failed to further drive the equilibrium of esterification, and tended to form ester bonds between the hydroxyl groups in cotton and the carboxyl groups in chitosan or citric acid. Overall, the surface change of chitosan film on fabrics from hydrophilic to hydrophobic was mainly caused by the decline in the contents of –NH_2_ in chitosan and –OH in cellulose [44]. This shift towards hydrophobicity for chitosan has also been found for the applications in the preparation of hydrophobic chitosan films via surface modification [45]. The hydrophobic coating of chitosan on the surfaces of treated samples (Figure 6d) prevents the water-soluble molecules of SCC from being dissolved in water again during the laundering process, thereby resulting in the improvement of the wash fastness of the treated samples.

ΔE of the SCC-dyed fabric was calculated after post-treatment to determine whether treatment resulted in a color change. The ΔE of all samples exceeded 2, indicating the treatment caused a visible color change [46]. Two reasons were responsible for color change. The acidic nature of the post-treatment solution caused the removal of dye molecules and the curing/cross-linking of the chitosan caused yellowing of the fabric.

### 3.4. Characterization

The characterization results of the four fabrics are presented in Figure 6.

The untreated cotton fibers had smooth surfaces (Figure 6a). The rough morphology of the fibers in Figure 6b–d confirms that chitosan was successfully loaded onto the cotton fabric after the pre-treatment and post-treatment processes.

The diffraction peaks at 2θ of 10.5° and 19.2° in Figure 6e can be assigned to the (0 0 1) and (1 0 0) lattices planes, and (1 0 1) and (0 0 2) lattice planes of chitosan, respectively. The diffraction peaks at 2θ of 16.6°, 22.6° and 34.5° can be assigned to the (1 0 1), (0 0 2) and (0 4 0) planes of cellulose, respectively [47]. No distinct diffraction peaks of chitosan can be observed from the diffraction patterns of the SCC-dyed fabric and the post-treated fabric. The reason is that during the dissolving and regeneration processes of chitosan, the crystallinity of chitosan was dramatically decreased. In addition, the amount of chitosan loaded onto the surface of the treated fabric was at a low level for the weight of fabric. Overall, no obvious deterioration of crystallinity of cellulose was observed after the cross-linking of chitosan.

The peak at 1729 cm^−1^ in Figure 6f, which can be assigned to the stretching vibration of C=O in the ester group [32], indicates the formation of ester bonds between chitosan and citric acid. The peaks at 1651 and 1557 cm^−1^ (partially enlarged spectra in Figure 6f) correspond to the C=O stretching vibration of amide I and the N–H deformation vibration of amide II, respectively [48,49]. As the chitosan used in this study was of a deacetylation degree of over 95%, and the loading amount of chitosan for the weight of fabric was at a low level, the peaks of amide groups at these two wavenumbers caused by the incomplete deacetylation were negligible. The appearance of peaks of amide I and II indicates that instead of forming the ester bonds with citric acid, the amine groups in chitosan molecules may also form amides with carboxyl groups in citric acid.

As the FTIR spectra only indicate that the esterification was possibly formed between chitosan and cellulose and provide no distinct trace of loading of chitosan on cotton, data from XPS scans were used as additional evidence to prove the loading of chitosan.

Peaks at 284.8, 400.5, 532.8 and 934.6 eV in Figure 6g correspond to the bands of C 1s, N 1s, O 1s and Cu 2p, respectively. The appearance of N 1s band in the wide scan spectrum of CTS coated fabric indicated the introduction of chitosan after the both the pre-treatment and post-treatment. The increase of N 1s band intensity (between CTS coated and the other two dyed samples) may be assigned to the porphyrin structure in the SCC absorbed by the treated sample during the dyeing process. The weak bands at 934.6 eV were introduced by the Cu^2+^ located in the center of porphyrin structures in SCC.

**Figure 7:**
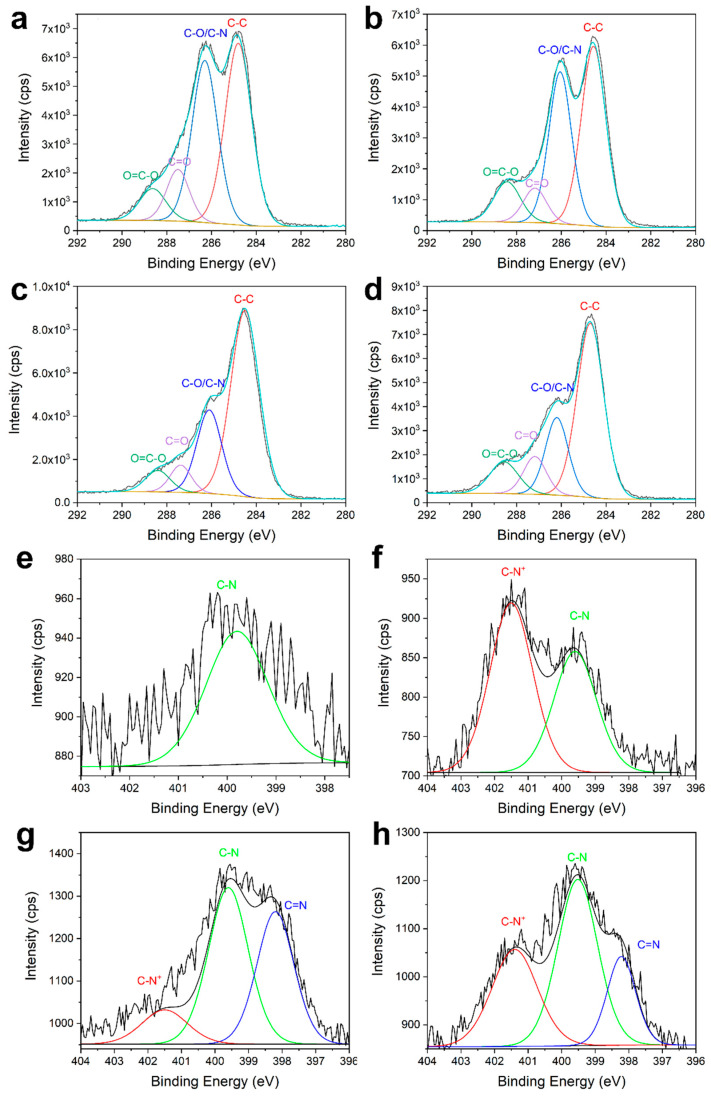
High-resolution C 1s spectra of the (**a**) greige fabric, (**b**) pre-treated fabric, (**c**) SCC-dyed fabric and (**d**) post-treated fabric; and high-resolution N 1s spectra of the (**e**) greige fabric, (**f**) pre-treated fabric, (**g**) SCC dyed fabric and (**h**) post-treated fabric.

The bands at 284.6, 286.1, 287.6 and 288.4 eV in the high-resolution C 1s spectra (Figure 7) can be assigned to C–C, C–O/C–N, C=O and O=C–O bonds, respectively. Compared with Figure 7a, the increase in intensity of the band at 288.4 eV in Figure 7b–d indicated the successful cross-linking of chitosan via the esterification. The sole band at 399.8 eV in Figure 7e corresponds to a C–N bond and can be assigned to the nitrogen-containing impurities in cellulose. The bands at 401.5 eV in Figure 7f–h can be assigned to C–N^+^, which was introduced to the fabric by the protonation of amines in chitosan, according to Cai et al. [50] and Ndong Ntoutoume et al. [51]. The bands at 398.2 eV in Figure 7g,h can be assigned to C=N bond in the porphyrin structure of SCC according to Rizzi et al. [52].

### 3.5. Handle and Flexibility

**Figure 8:**
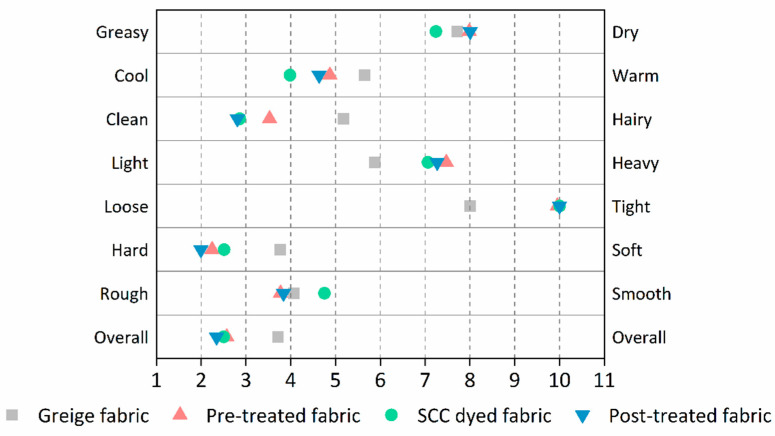
Handle of the samples.

The results of handle evaluation of the four samples are presented in Figure 8.

As shown in Figure 8, seven indexes were given to characterize the different aspects of handle. The value of each index varied on a scale of 0 to 10, representing the index’s tendency towards the two opposite criteria. An overall index, based on the seven individual indexes, was given to summarize the overall handle of the sample. It can be seen from Table 2 that loading of chitosan onto the surface of fabric leads to an increase of fabric weight. The gaps between the fibers and loops formed by the yarns were filled with chitosan (Figure 6b–d). This resulted in an increased fabric stiffness, which in turn led to a decreased fabric looseness and softness. However, the porous structure of chitosan coatings on the surfaces of treated fabric, which was caused by the evaporation of chitosan solution during the curing process, led to a rougher surface of the treated fabric. Overall, the handle of the treated fabric worsened due to the cross-linking of chitosan.

**Figure 9:**
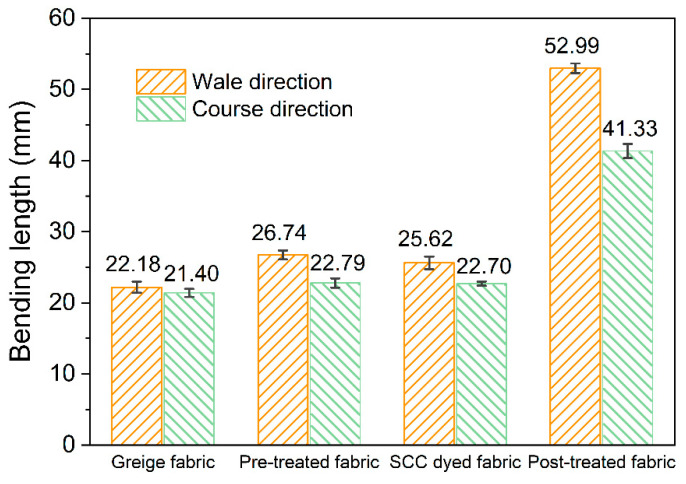
Flexibility of the four samples from the perspective of bending length.

The bending lengths of the four samples along their wale and course directions are presented in Figure 9 to reflect the flexibility properties of the samples. All the bending lengths of the four samples along their wale directions were higher than those corresponding to their course directions. This difference in the bending length between the two directions can be attributed to the nature that the tension in the inner surfaces of loops is higher than that of in the yarns along the wale direction, which indicates that the single jersey knitted fabric is more likely to roll along its course direction [53]. The bending lengths of the pre-dyed fabric, SCC-dyed fabric and post-treated fabric were all higher than that of the greige fabric, indicating that the flexibility of the treated fabric worsened due to the formation of chitosan layer on the cotton fabric. Specifically, the bending length of the post-treated fabric almost doubled compared with the pre-treated fabric. This dramatic change in flexibility was caused by the thicker chitosan layer cured on the dyed fabric during the post-treatment. It is also notable that the bending lengths of the SCC-dyed fabric slightly decreased compared with the pre-treated fabric. This decline in bending length can be attributed to the hydrolysis of the ester bonds between carboxyl groups in citric acid and the hydroxyl groups in chitosan or cellulose (Figure S5) during the dyeing process. Both the results from the handle and results from the flexibility tests confirmed that the mechanical properties of the cotton fabric worsened after the cross-linking treatment. Specifically, a higher concentration of chitosan solution will lead to worse handle and flexibility properties of the treated fabric.

## 4. Conclusions

Cross-linking of chitosan onto cotton fabric via the cross-linking agent of citric acid can improve the exhaustion of SCC, thereby increasing the K/S of SCC-dyed fabric. The adsorption kinetics of SCC can be fitted with the pseudo-second-order model; the calculated and experimental dye absorption per unit weight of fabric were dramatically improved. The isotherms of SCC adsorption can be fitted with the Langmuir model. The calculated maximum *q_e_* reaches 19.8 g/kg after the cross-linking of chitosan, while the maximum *q_e_* of the untreated control is 4.5 g/kg. Under the optimal treating conditions, the dye exhaustion of the treated fabric reached 96.4%, while the dye exhaustion of untreated control was 22.7% with 1% o.w.f. dye concentration.

Post-treating the SCC-dyed fabric with a high-concentration (≥20 g/L) chitosan solution via the pad method is a feasible way to improve the wash fastness of the fabric. When the dyed fabric was post-treated with 20 g/L chitosan solution at a pick-up rate of 100 ± 5% and cured at 180 °C for 100 s, and thoroughly rinsed, its wash fastness was improved from 3 to 5. SEM images show the porous and rough morphology of the chitosan layer deposited on the surfaces of the treated fabrics. FTIR results confirmed the esterification between the carboxyl groups of citric acid and the hydroxyl groups of chitosan. XPS spectra verified the successful loading of chitosan onto the cotton fabric. XRD patterns show that cross-linking of chitosan has no distinctive effect on the crystallinity of cellulose.

In summary, the color fastness properties of the cotton fabric dyed with SCC can be improved from 3 to 4 on the scale according to Method 3 in ISO 105 B-02 with the inevitable deterioration of the dyed fabric’s handle. The dyed fabric may be found in applications in apparel which require stiff handles or applicational textiles, such as coats and curtains.

## Figures and Tables

**Table 1:** Treatment chemicals used for the 4 fabrics treated under different conditions before dyeing with SCC.

Sample No.	1	2	3	4
0.2 g chitosan	/	/	/	✓
2 g citric acid	/	/	✓	✓
Washed with 10 g/L sodium citrate	/	✓	✓	✓

**Table 2:** K/S and dye exhaustion of fabrics under different pre-treatment conditions and dye concentrations.

Sample No.	Factor 1 Chitosan (g/L)	Factor 2 Dye Concentration (% o.w.f.)	Factor 3 Curing Time (s)	Factor 4 Pick-up (%)	Rate of Weight Increase (R_w_ %)	K/S	Dye Exhaustion (%)
1	4	1.5	100	100	3.1	3.2	28.9
2	12	1	60	100	4.7	11.9	96.4
3	8	1.5	60	85	3.9	7.3	47.2
4	12	1.5	80	70	5.4	3.2	31.9
5	4	0.5	60	70	1.0	3.1	43.7
6	12	0.5	100	85	5.6	3.2	38.0
7	8	1	100	70	3.4	3.2	37.1
8	8	0.5	80	100	4.7	3.4	41.2
9	4	1	80	85	1.7	3.1	38.8

**Table 3:** Wash fastness values and contact angles of the post-treated fabrics, and the color change caused by each treatment.

Sample No.	Factor 1	Factor 2	Factor 3	Wash Fastness	Light Fastness	Rubbing Fastness		Contact Angle (°)	Color Change (ΔE)
	Chitosan (g/L)	Curing Time (s)	Pick-up (%)			Dry	Wet		
Control	0	0	0	3	3	4-5	1–2	95	2.3
1	20	100	100	5	3–4	5	3–4	126	2.5
2	40	60	100	4	3–4	5	3–4	126	3.0
3	20	60	70	4–5	3–4	5	3–4	139	2.1
4	30	60	85	4	3–4	5	3–4	120	2.7
5	40	80	70	4-5	4	5	3–4	132	2.9
6	40	100	85	4-5	4	5	4	144	2.8
7	30	80	100	4-5	4	5	3–4	128	2.9
8	30	100	70	5	4	5	4	124	2.8
9	20	80	85	4-5	3–4	5	3–4	133	2.9

## Supplementary Materials

The following are available online at https://www.mdpi.com/1996-1944/13/23/5365/s1. Figure S1: Diagram of the dipping and padding process, Figure S2: Yellowing of the pre-treated fabric, Figure S3: Captured contact angle images for green samples C (control) and 1–9 (samples 1–9 in the orthogonal design), Figure S4: High-resolution Cu 2p spectra of (a) SCC-dyed fabric and (b) post-treated fabric, Figure S5: Scheme of reaction of cross-linking chitosan to cellulose by citric acid, Table S1: Color strength (K/S) and CIE color coordinates of three pieces of SCC-dyed fabric obtained under the optimal pre-treatment conditions and dye concentration, Table S2: Color strength (K/S) and CIE color coordinates of the SCC-dyed fabric with and without the post-treatment, Table S3: Color strength (K/S) and CIE color coordinates of the untreated control and the 9 samples which have been subject to the post-treatment.
