# Supplementary material for: In Situ Synthesis of a Double-Layer Chitosan Coating on Cotton Fabric to Improve the Color Fastness of Sodium Copper Chlorophyllin
Source: Materials (Basel). 2020 Nov 26;13(23):5365. doi: 10.3390/ma13235365 (PMC7730442; doi:10.3390/ma13235365)
Supplement: Supplementary file 1 [file materials-13-05365-s001.pdf]

Table S1. Color strength (K/S) and CIE color coordinates of three pieces of SCC dyed fabric obtained under the optimal pre-treatment conditions and dye concentration.

Sample No.	K/S	L*	a*	b*	Dye exhaustion
1	12.1	41.0	-9.6	10.4	93.9
2	11.4	41.6	-9.6	10.1	90.8
3	12.2	40.6	-9.6	10.4	96.7

Three SCC dyed fabrics were prepared following the optimal pre-treatment conditions and the same dye concentration listed in Table 2. The dye exhaustion of the three samples were both higher than 90 % (Table S1). The K/S and CIE color coordinates were both very close to those of Sample 2 listed in Table 2. These results verify the reproducibility of the optimal pre-treatment conditions.

Table S2. Color strength (K/S) and CIE color coordinates of the SCC dyed fabric with and without the post-treatment.

Sample	K/S	L*	a*	b*
Without post-treatment	11.9	41.2	-9.4	10.4
With post treatment	9.85	37.9	-5.0	9.6

Table S3 Color strength (K/S) and CIE color coordinates of the untreated control and the 9 samples which have been subject to the post-treatment.

Sample No.	K/S	L*	a*	b*
Untreated control	11.9	41.2	-9.4	10.4
1	10.9	41.4	-7.0	10.5
2	9.9	42.2	-7.1	9.7
3	11.4	40.5	-7.5	10.2
4	10.9	42.1	-7.7	10.4
5	11.4	39.5	-6.8	9.0
6	10.7	40.7	-6.6	9.8
7	12.1	39.1	-7.0	10.1
8	10.8	41.9	-7.2	10.0
9	10.1	42.1	-7.0	10.1

Subjecting the SCC dyed fabric to the post-treatment will cause color change of the treated fabric. Specifically, the color change was generated in two processes: immersion of the dyed fabric in the chitosan solution and curing of the chitosan loaded on the surface of the dyed fabric. A small proportion of SCC molecule were dissolved again in the chitosan solution when the dyed fabric was immersed in the chitosan solution. Another small amount of SCC molecule may decompose during the curing process. The former can be confirmed by the decrease in K/S (Table S2 and S3) while the latter can be confirmed by the decrease in a* (Table S2 and S3).

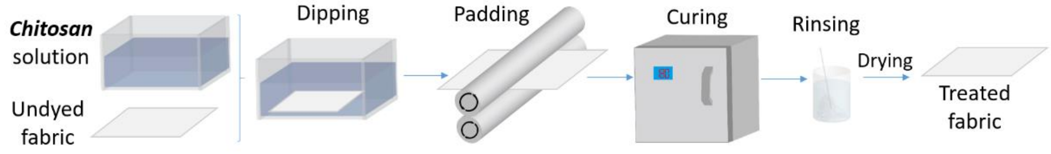

Figure S1 Diagram of dipping and padding process

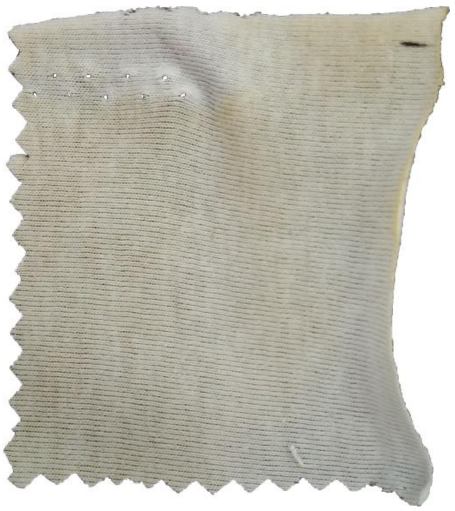

Figure S2 Yellowing of the pre-treated fabric

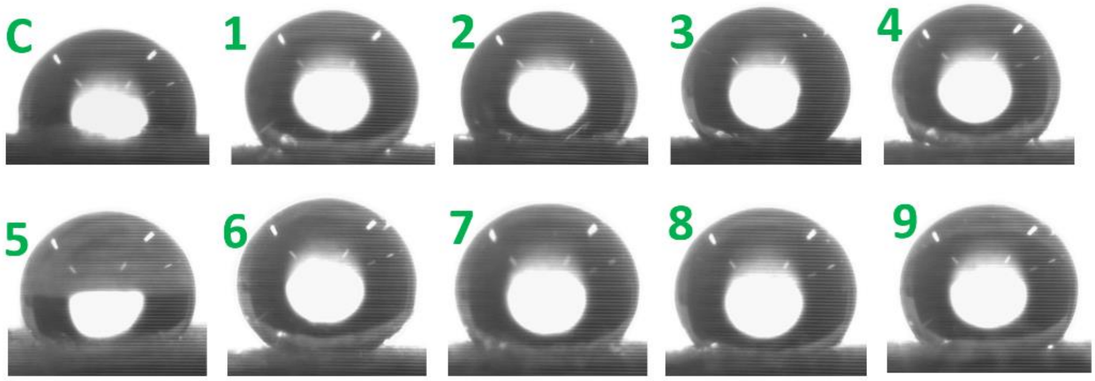

Figure S3 Captured contact angle images for green samples C (control) and 1-9 (Samples 1-9 in the orthogonal design)

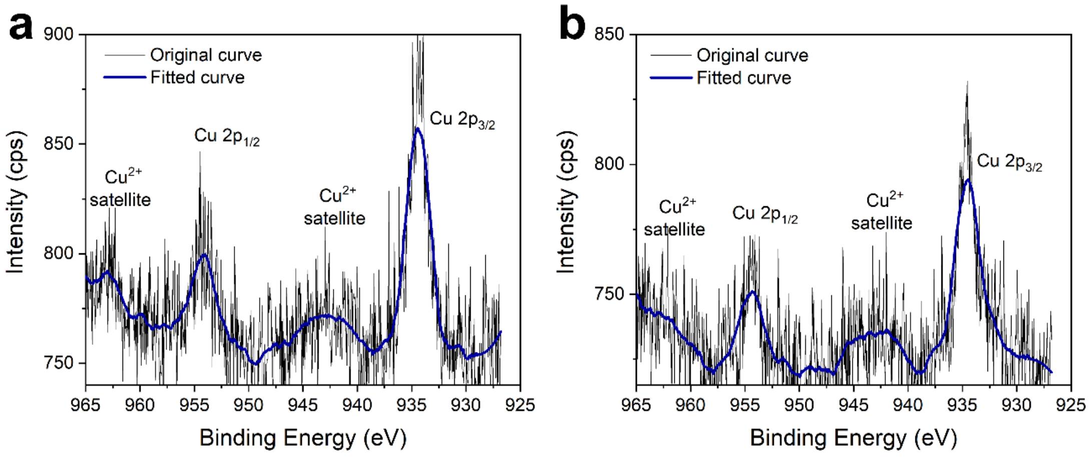

Figure S4 High-resolution Cu 2p spectra of **a** SCC dyed fabric and **b** Post-treated fabric

Figure S4 a and b show the high-resolution Cu 2p spectra of SCC dyed fabric and post-treated fabric, respectively. The bands at 934.7 eV, 953.8 eV can be assigned to the peak of Cu $2p_{3/2}$ and Cu $2p_{1/2}$, respectively. The bands at 962.8 eV and 943.1 eV can be assigned to the shake-up satellite peaks of Cu^{2+}. The binding energy of these characteristic peaks of Cu 2p are in accordance with the XPS data reported by Zhou et al. (2019).

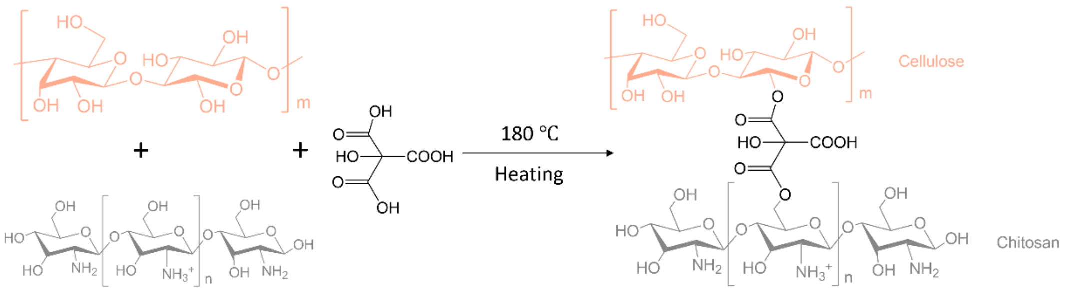

Figure S5 Scheme of reaction of cross-linking chitosan to cellulose by citric acid

References

Zhou, S., He, H., Guo, W., Zhu, H., Xue, F., Cheng, M., . . . Wang, S. (2019). Structural design of a high sensitivity biomass cellulose-based colorimetric sensor and its in situ visual recognition mechanism for Cu^{2+}. *Carbohydrate Polymers*, 219, 10.
